# Curcumin’s Beneficial Effects on Neuroblastoma: Mechanisms, Challenges, and Potential Solutions

**DOI:** 10.3390/biom10111469

**Published:** 2020-10-22

**Authors:** Kevin Zhai, Aranka Brockmüller, Peter Kubatka, Mehdi Shakibaei, Dietrich Büsselberg

**Affiliations:** 1 Department of Physiology and Biophysics, Weill Cornell Medicine-Qatar, Education City, Qatar Foundation, Doha 24144, Qatar; kez4003@qatar-med.cornell.edu; 2 Musculoskeletal Research Group and Tumor Biology, Chair of Vegetative Anatomy, Institute of Anatomy, Faculty of Medicine, Ludwig-Maximilian-University Munich, 80336 Munich, Germany; aranka.brockmueller@campus.lmu.de (A.B.); mehdi.shakibaei@med.uni-muenchen.de (M.S.); 3 Department of Medical Biology, Jessenius Faculty of Medicine, Comenius University in Bratislava, 03601 Martin, Slovakia; peter.kubatka@uniba.sk

**Keywords:** curcumin, neuroblastoma, apoptosis, natural substances, oncology, cancer prevention

## Abstract

Curcumin, a natural polyphenolic compound derived from the South Asian turmeric plant (*Curcuma longa*), has well-characterized antioxidant, anti-inflammatory, anti-protein-aggregate, and anticancer properties. Neuroblastoma (NB) is a cancer of the nervous system that arises primarily in pediatric patients. In order to reduce the multiple disadvantages and side effects of conventional oncologic modalities and to potentially overcome cancer drug resistance, natural substances such as curcumin are examined as complementary and supportive therapies against NB. In NB cell lines, curcumin by itself promotes apoptosis and cell cycle arrest through the suppression of serine–threonine kinase Akt and nuclear factor kappa of activated B-cells (NF-κB) signaling, induction of mitochondrial dysfunction, and upregulation of p53 and caspase signaling. While curcumin demonstrates anti-NB efficacy in vitro, cross-validation between NB cell types is currently lacking for many of its specific mechanistic activities. Furthermore, curcumin’s low bioavailability by oral administration, poor absorption, and relative insolubility in water pose challenges to its clinical introduction. Numerous curcumin formulations, including nanoparticles, nanocarriers, and microemulsions, have been developed, with these having some success in the treatment of NB. In the future, standardization and further basic and preclinical trials will be required to ensure the safety of curcumin formulations. While the administration of curcumin is clinically safe even at high doses, clinical trials are necessary to substantiate the practical efficacy of curcumin in the prevention and treatment of NB.

## 1. Natural Substances in the Prevention and Treatment of Neuroblastoma

Neuroblastoma (NB) is a solid pediatric tumor of the nervous system that arises from neural progenitor cells. Neuroblastoma occurs mostly in the abdominal region, especially in and around the adrenal gland. Tumors may also form in the central nervous system (CNS) and the thoracic and pelvic regions [1]. The annual epidemiological occurrence of NB varies regionally, with approximately 13 cases per million children in Germany and 8.3 cases per million children in Argentina [2,3]. Five-year survival rates also vary, with 47% survival among 971 patients in Argentina, 48% among 1094 patients in Europe, and 57% among 265 patients in the United States [3,4].

Notably, the risk posed by NB depends on numerous factors, including patient age, inflammation, protein aggregation, tumor localization or metastasis, and genetic disposition. To take these factors into account, the International Neuroblastoma Risk Group Staging System establishes four stages of NB progression (L1, L2, M, and MS) and four risk levels (very low, low, intermediate, and high) [5,6]. One of the major risk factors in NB is the amplification of the N-myc proto-oncogene protein (MYCN), which promotes NB tumorigenesis. Other risk factors include patient age and the deletion of a portion of chromosome 11 (11q aberration). Both MYCN amplification and 11q aberration are markers of poor clinical prognosis [7].

A variety of human and rodent cell lines are utilized to account for NB risk factors and other genetic variables within in vitro studies. These include the MYCN-amplified human cell lines IMR-32, Kelly, LAN5, SK-N-Be(2), SMS-KAN, and NUB-7. Among these lines, IMR-32, Kelly, SK-N-Be(2), and SMS-KAN exhibit 11q aberrations. In contrast, non-MYCN-amplified human cell lines include L-AN-6, SH-SY5Y, SK-N-AS, and SK-N-SH; of these, L-AN-6, SH-SY5Y, and SK-N-AS cells exhibit 11q aberrations [8]. Finally, rodent cell lines include the murine Neuro2a line and the rat B50 line.

Conventional treatments for NB include chemotherapy, radiation therapy, surgical tumor resection, and combinations thereof. Surgery alone may sufficiently ablate low-risk NB; supporting chemotherapy with carboplatin, etoposide, cyclophosphamide, and doxorubicin may occur in some cases [9]. Reduced-dose chemotherapy with the drugs mentioned above can similarly control intermediate-risk NB [10]. In contrast, a treatment regimen for high-risk NB could include the following: (1) induction chemotherapy with cisplatin, carboplatin, etoposide, vincristine, and cyclophosphamide; (2) surgical removal of the primary tumor, with or without additional topotecan, vincristine, and doxorubicin therapy; (3) consolidation chemotherapy utilizing busulfan and melphalan, or carboplatin, etoposide, and melphalan; (4) stem cell rescue; (5) radiation therapy; and (6) maintenance chemotherapy, with or without immunotherapy [11]. These often complex and multifaceted conventional treatment regimens are clinically trialed and effective. However, they also involve some significant disadvantages. Chemotherapy, for instance, can induce potentially harmful side effects, including toxicity and myelosuppression [10]. Surgical interventions, on the other hand, are invasive and can lead to incomplete tumor resection, requiring further chemo- and radiotherapy and possibly stem cell transplantation [12].

In attempting to address the disadvantages posed by conventional oncologic therapies, the anticancer properties of natural substances have been widely investigated. Natural substances are naturally occurring chemical compounds, many of which are dietary. Some of these compounds can support cancer prevention and therapy or overcome cancer drug resistance with few or no side effects. Flavonoids, for example, suppress cancer through modulation of extracellular matrix (ECM) proteins, inhibition of the epithelial–mesenchymal transition (EMT), and inhibition of cancer cell metabolism [13,14]. Plant phenolic acids and lichens upregulate apoptosis, inhibit proliferation and metastasis, and induce cell cycle arrest, while vanilloids such as capsaicin exert anticancer effects through the modulation of intracellular calcium signaling [15,16,17]. Significantly, several natural substances are effective against NB cells in vitro. Isoliquiritigenin, a flavonoid, induces necrotic cell death and cell cycle arrest and downregulates intracellular adenosine triphosphate (ATP) in SH-SY5Y cells [18]. Another flavonoid, rutin, downregulates the antiapoptotic protein B-cell lymphoma 2 (Bcl-2) and MYCN, upregulates tumor necrosis factor alpha (TNF-α), and induces cell cycle arrest and apoptosis in LAN-5 cells [19]. Juniper berry extract, which also includes flavonoids, upregulates p53 and induces endoplasmic reticulum (ER) stress and apoptosis in SH-SY5Y cells [20]. Furthermore, resveratrol, a natural polyphenol found in berries, grapes, and nuts, demonstrates several anticarcinogenic effects on NB cells—it increases cytotoxicity, inhibits the cell cycle, induces apoptosis, and reduces the overall growth rate. Initiator and effector caspase activation, inhibition of Bcl-2, and downregulation of p21 are known mechanisms [21,22]. Finally, curcumin, a plant-derived non-flavonoid substance, exhibits a wide range of anticancer properties toward NB cell lines, which will be investigated in this review.

In evaluating the potential of curcumin in preventing and treating NB, it should be emphasized that in contrast to most known tumors, the tumor suppressor protein p53 occurs as a wild type in almost all NB tumors [23]. Initially, p53 is primarily localized to the cytosol, where it is irregularly stabilized, accumulated, and overexpressed [24,25]. This is a typical mechanism for the functional inactivation of p53 in NB [24,25,26]. However, the p53 signaling pathway can be activated in several NB cell lines despite its cytoplasmic localization and nuclear exclusion [26,27,28]. In addition, the expression of p53 in malignant NB cells, as opposed to non-malignant NB cells, is significantly downregulated at the physiological level [24,25,29]. This underscores the possibility that these cells can undergo apoptosis [30,31]. Interestingly, the p53 transformation process in NB cells is reversible, suggesting that it could represent a therapeutic target. Malignant NB cells are often resistant to typical radiation and chemotherapy treatments. The upregulation of transcription factors such as NF-κB and activator protein 1 (AP-1) in tumors is partly responsible for this increased resistance [32,33]. Curcumin, a strong and natural NF-κB inhibitor [34,35,36,37,38,39,40], significantly reduces cell survival and modulates the expression of apoptosis-related genes in human NB cells in combination with radiation therapy [32]. Notably, p53-dependent cell death in various tumor cells can be specifically and effectively activated by treatment with the naturally occurring polyphenol curcumin [41].

## 2. Structure and Biological Properties of Curcumin

Curcumin ((1E,6E)-1,7-bis(4-hydroxy-3-methoxyphenyl)hepta-1,6-diene-3,5-dione) is derived from the rhizome of the turmeric plant (*Curcuma longa*). Turmeric is native to South Asia (e.g., India, China, and Indonesia) and is cultivated for its traditional medicinal properties [42].

**Figure 1:**
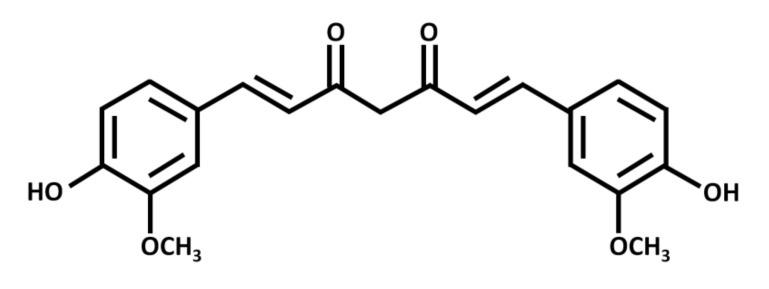
Chemical structure of curcumin, a polyphenolic chemical constituent of turmeric with antioxidant, anti-inflammatory, and anticancer effects. Curcumin is a beta-diketone compound containing two substituted aromatic rings linked by a seven-carbon chain. Each aromatic ring has one hydroxy and one methoxy group.

Curcumin exists as a yellow-orange solid, with a molecular weight of 368 g/mol and a melting point of 183 °C. Chemically, it is a polyphenolic compound with two aromatic rings, each with one hydroxy and one methoxy substituent (Figure 1). A seven-carbon chain with two α-β unsaturated carbonyl groups (which are subject to tautomerization) links the rings [43].

Commercially available curcumin contains three primary components, namely diferuloylmethane (the most abundant and active component of turmeric, at 82%) and its derivatives demethoxycurcumin (15%) and bisdemethoxycurcumin (3%); these are collectively referred to as “curcuminoids” [44,45]. Curcuminoids have the potential to treat various diseases through the modulation of molecular signaling targets, including transcription factors (e.g., NF-κB, AP-1, ß-catenin, and peroxisome proliferator-activated receptor), enzymes (e.g., COX-2, 5-LOX, and iNOS), and pro-inflammatory cytokines (e.g., TNF-α, IL-1, and IL-6) [44,46,47,48,49,50,51].

The biological and physiological properties of curcumin have been extensively examined and reviewed in the context of neurodegenerative and inflammatory diseases, such as Alzheimer’s disease and dementia. Curcumin exhibits notable antioxidant activities through iron chelation, inhibition of lipid peroxidation, and scavenging of reactive oxygen species (ROS) [52]. The compound also has anti-inflammatory properties in acute and chronic inflammation [53]. Beyond these functions, curcumin is widely examined as an anticancer agent. Orally administered curcumin—alone and in combination with conventional chemotherapeutics—has been clinically trialed in pancreatic, breast, and prostate cancer patients [54,55,56,57]. Curcumin also demonstrates oncologic effects by itself on murine glioblastoma xenografts and in combination with the chemotherapeutic 5-fluorouracil on colorectal cancer cells [49,58,59,60].

Although curcumin has many positive effects on health, it is sometimes criticized for its low bioavailability—a result of its low absorption and rapid metabolism by the body. However, its bioavailability can be enhanced through combination with adjuvants. Indeed, piperine, a component of black pepper, is suitable for this purpose [61,62,63]. Notably, while curcumin’s anticancer properties are frequently reported, its membrane transport mechanisms remain mostly uncharacterized. Molecular dynamics simulations and solid-state nuclear magnetic resonance experiments indicate that curcumin can insert itself into plasma membranes [64,65]. However, specific transport mechanisms for the nervous system and NB cells are unclear at this time.

Despite these shortcomings, curcumin is a major component of numerous spices and foods, and is well tolerated in relatively high concentrations as part of daily diets without side effects. As such, it has high potential as a putative chemopreventive or therapeutic agent [66].

## 3. Description of the Study

### 3.1. Rationale and Aims of the Study

A vast body of preclinical literature is available on the anticancer properties of curcumin. Promising results have arisen from pancreatic and colon cancer cells, and within the CNS in glioblastoma and brain tumor cells [67]. Within the previous decade, clinical trials have substantiated the potential oncologic applications of curcumin for several common cancer types (pancreatic, prostate, and breast cancers). Moreover, clinical trials have also tested the anticancer properties of curcumin in the nervous system. Its effects on glioblastoma cell lines have been widely documented and extensively reviewed. However, the literature relating curcumin and NB, a rare malignancy with poor prognosis, remains comparatively sparse.

As such, this review explores curcumin’s viability as a natural supportive therapy for the prevention and treatment of NB. It first summarizes existing preclinical trials of curcumin on NB cell lines and presents overarching pathways through which curcumin may exert oncologic effects. This study then notes the synergistic and combinatorial effects of curcumin with other natural substances, chemotherapeutics, and radiotherapy on NB. Moreover, the differential effects of curcumin on various NB cell lines and types are explored. Finally, this review discusses the current challenges involved in curcumin delivery and presents some potential solutions and improvements.

### 3.2. Study Methodology

Core literature for this review was obtained from PubMed and Google Scholar, using the keywords “curcumin,” “neuroblastoma,” and “apoptosis.” A PubMed search with these keywords yielded 42 literature results. Approximately 20 of these studies were utilized for this review. Studies including both curcumin and NB but concerning subjects other than oncology (e.g., Alzheimer’s disease, or neurodegeneration) were excluded. Further literature (approximately 10 recent studies) on the bioavailability and absorption of curcumin was obtained using the keywords “curcumin”, “bioavailability”, “nervous system”, or “brain delivery”.

## 4. Curcumin as an Anticancer Agent in NB

Preclinical trials demonstrate that curcumin exerts a range of oncologic effects on NB cell lines. These include the induction of apoptosis and cell cycle arrest, downregulation of proliferative signaling pathways, inhibition of cell migration, and disruption of cellular glucose metabolism (Table 1).

### 4.1. Apoptosis and Cell Cycle Arrest

**Figure 2:**
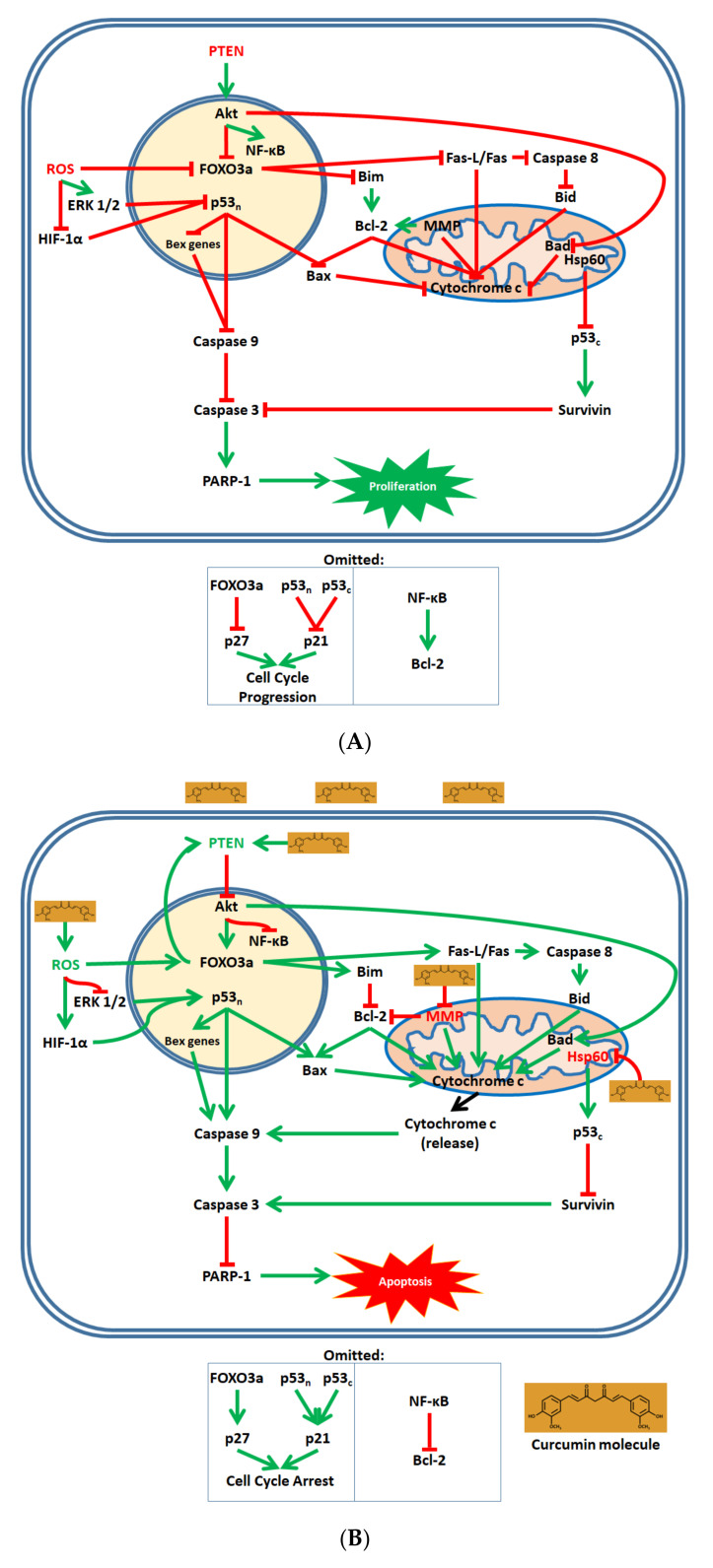
Curcumin, as a multitarget compound, induces apoptosis and cell cycle arrest in neuroblastoma (NB) cells through the modulation of PTEN–Akt, NF-κB, and p53 signaling; mitochondrial dysfunction; and caspase activation. (**A**) Intracellular signaling mechanisms within NB cells favor proliferation. The proliferative Akt, NF-κB, and ERK 1/2 pathways, as well as the antiapoptotic Bcl-2 and Survivin proteins, are active and upregulated. Apoptotic signals via mitochondrial dysfunction, cytochrome c release, p53, and caspases are downregulated. Cyclin-dependent kinase inhibitors (p21 and p27) are inactive, allowing for cell cycle progression. (**B**) Curcumin modulates intracellular signaling in NB cells in favor of apoptosis and cell cycle arrest. Curcumin upregulates PTEN, which in turn downregulates Akt and NF-κB and upregulates FOXO3a and Fas pathway signaling. Moreover, curcumin-induced ROS generation elevates intracellular ROS levels and supports both FOXO3a and p53_n_ signaling. Together, p53_n_, Fas pathway, and Akt signaling, along with mitochondrial membrane potential (MMP) depolarization, promote the release of cytochrome c from the mitochondria into the cytosol, where it supports caspase activation. Mitochondrial dysfunction also involves the downregulation of Hsp60, which ultimately leads to the downregulation of Survivin—a protein that ordinarily inhibits caspase activity. Caspase 3 cleaves PARP-1, causing apoptosis, while FOXO3a and p53 activate cyclin-dependent kinase inhibitors, which induce cell cycle arrest.

In cancerous cells, proliferative and pro-survival effectors such as the serine–threonine kinase Akt, nuclear factor kappa light-chain enhancer of activated B-cells (NF-κB), Bcl-2, and Survivin are active, while apoptotic effectors such as cytochrome c, p53, and caspases are suppressed. Curcumin shifts the intracellular signaling mechanisms in NB to upregulate apoptosis and cell cycle arrest [41]. Mechanistically, curcumin promotes apoptosis through the downregulation of Akt and NF-κB signaling, induction of mitochondrial dysfunction, upregulation of p53, and activation of caspases (Figure 2).

Upon its in vitro application to NB cells, curcumin firstly modulates phosphatase and tension homolog (PTEN)–Akt signaling. Curcumin upregulates PTEN, which downregulates Akt and reduces Akt translocation from the cytosol to the nucleus [68]. Downregulation of Akt, in turn, leads to (1) downregulation of NF-κB; (2) upregulation of the pro-apoptotic mitochondrial protein Bad and induction of its translocation from the cytosol to the mitochondria; and (3) upregulation of the forkhead box O3a (FOXO3a) protein and induction of its nuclear translocation from the cytosol [68,69,70]. FOXO3a consequently upregulates the pro-apoptotic Bcl-2-interacting mediator of cell death (Bim), as well as the Fas ligand (Fas-L). Elevated levels of Fas-L induce the Fas pathway, in which Fas-L and Fas activate caspase 8, a proteolytic enzyme that cleaves the BH3-interacting domain death agonist (Bid) protein into its active form [68,71] (see Figure 2).

In inducing mitochondrial dysfunction, curcumin depolarizes the mitochondrial membrane potential (MMP), leading to the downregulation of Bcl-2 [72]. The upregulation of Bim and downregulation of NF-κB, as previously discussed, also contribute to the downregulation of Bcl-2 [69,73]. In turn, the downregulation of Bcl-2 causes the upregulation of the pro-apoptotic Bcl-2-associated X protein (Bax) [72]. Together, the upregulation of Bax, Fas-L, Bid, and Bad; the depolarization of the MMP; as well as the downregulation of Bcl-2 all contribute to the release of cytochrome c from the mitochondria into the cytosol [70,71,72,74]. Additionally, curcumin downregulates the mitochondrial heat shock protein Hsp60 [68].

Concurrently with the modulation of PTEN–Akt signaling and induction of mitochondrial dysfunction, curcumin highly regulates pro-apoptotic p53 signaling in NB cells. The increase of intracellular ROS levels by curcumin leads to the downregulation of the proliferative extracellular-signal-regulated kinases (ERK) 1/2 and the upregulation of hypoxia-inducible factor 1 alpha (HIF-1α) [72,75]. HIF-1α and downregulated ERK 1/2 consequently upregulate nuclear p53 (p53_n_) [75,76]. In turn, p53_n_ further upregulates Bax and induces the pro-apoptotic brain-expressed X-linked (Bex) genes [75,77]. Moreover, downregulation of mitochondrial Hsp60 allows for the upregulation of cytosolic p53 (p53_c_), which in turn downregulates the pro-survival protein Survivin [78].

At the end of the pro-apoptotic axis, cytochrome c release, Bex genes, and p53_n_ activate caspase 9 [71,75]. Caspase 9 activates caspase 3; Survivin, which ordinarily inhibits apoptosis by downregulating caspase 3, is itself downregulated and does not perform this function [79]. Finally, caspase 3 cleaves poly(ADP-ribose) polymerase 1 (PARP-1), leading to apoptosis.

In addition to apoptosis, curcumin also promotes cell cycle arrest in various NB cell lines. FOXO3a upregulates the cyclin-dependent kinase inhibitor p27, while p53 upregulates the cyclin-dependent kinase inhibitor p21 [68,80]. Together, p21 and p27 induce cell cycle arrest [68,77].

Interestingly, curcumin activates the degradation of amyloidogenic proteins, including the amyloid β precursor protein and α-synuclein, via translocation of the transcription factor EB–autophagy signaling pathway. This activity is regulated by the inhibition of GSK-3β signaling, which thereby increases antioxidant gene expression in human NB cells [81]. Bavisotto and co-workers demonstrated that curcumin decreases heat shock protein (Hsp60) levels and upregulates Hsp60 mRNA expression, and thereby causes apoptosis. Curcumin downregulates the ubiquitination and nitration of Hsp60 and increases chaperonin levels, indicating that it may disrupt NB progression through a protective pathway involving chaperonin [82].

### 4.2. Effects on NB Cell Migration and Glucose Metabolism

Beyond the induction of apoptosis and cell cycle arrest, curcumin modulates NB cell migration and glucose metabolism. Curcumin upregulates TIMP metallopeptidase inhibitor 1 (TIMP-1), which downregulates ECM matrix metalloproteinase 2 (MMP-2), and thereby inhibits cell migration [80]. In fact, D’Aguanno and colleagues demonstrated that cell organization and assembly are inhibited because curcumin downregulates necessary proteins [83]. Additionally, curcumin downregulates the glucose-metabolism-related proteins lactate dehydrogenase (LDH), aldolase (ALDOA), triosephosphate isomerase (TPI1), glyceraldehyde-3-phosphate dehydrogenase (GAPDH), and enolase (ENO1) [83]. As such, curcumin inhibits the following conversions: pyruvate to lactate by LDH; fructose 1,6-biphosphate (FBP) to dihydroxyacetone (DHAP) and glyceraldehyde 3-phosphate (G3P) by ALDOA; DHAP to G3P by TPI1; G3P to 1,3-bisphosphoglycerate (1,3-BPG) by GAPDH; and 2-phosphoglycerate (2-PG) to phosphoenolpyruvate (PEP) by ENO1. Since evidence of the Warburg effect has been found in NB, the associated mechanisms could represent a further point of targeting by curcumin. Curcumin can suppress the Warburg effect in other cancer cell lines, and thus influence their glucose metabolism [84,85].

**Table 1:** Anticancer effects of pure curcumin on NB cell lines. Curcumin modulates genes and proteins that regulate proliferation, apoptosis, cell cycle progression, cell motility, and intracellular glucose metabolism. Downregulations, decreases, and inhibitions are in bold.

Effect	Cell Line	Effective Concentration/Dosage	Source
**Decreases proliferation**	Neuro2a	10, 25, 50 µM	[75]
	SH-SY5Y		[74]
	SK-NBE2c		[74]
	LAN5		[74]
	HTLA-230		[74]
	GI-LI-N		[74]
	IMR-32		[74]
**Decreases clonal growth/colony formation**	GI-LI-N	20 µM	[74]
Increases cell death/decreases viability	Neuro2a	10, 25, 50 µM	[75]
	LAN5	15, 20 µM	[68]
	LAN5	0.001, 0.01, 0.1, 1, 10, 100 µM	[86]
	LAN5	3.125, 6.25, 12.5, 25, 50, 100, 200 µM	[82]
	LAN5	10, 20, 40, 80 µM	[77]
	SK-N-SH	0.001, 0.01, 0.1, 1, 10, 100 µM	[86]
	SK-N-SH	1, 5, 10, 50, 100, 500 µM	[87]
	Kelly	0.001, 0.01, 0.1, 1, 10, 100 µM	[86]
	IMR-32	100 µM	[72]
	IMR-32	10, 25, 50, 100 µM	[77]
	IMR-32	10, 100 µg/mL	[88]
	SMS-KAN	100 µM	[72]
	SK-N-AS	100 µM	[72]
	SK-N-AS	10, 25, 50, 75, 100 µM	[69]
	LA-N-6	100 µM	[72]
	SH-SY5Y	5, 10, 20, 50 µM	[80]
	SH-SY5Y (WT)	10, 20, 40, 80 µM	[83]
	SH-SY5Y (DDP)	10, 20, 40, 80 µM	[83]
	NUB-7	5, 10, 20, 40, 80 µM	[77]
	SK-N-Be(2)	10, 25, 50, 100 µM	[77]
	SK-N-Be(2)	25, 50, 75, 100 µM	[69]
	SK-N-MC		[89]
	SK-N-FI	5, 10, 50, 100, 500 µM	[87]
Increases apoptosis	Neuro2a	50 µM	[75]
	LAN5	15, 20 µM	[68]
	LAN5	12.5, 25 µM	[82]
	Kelly	10, 100 µM	[86]
	IMR-32	100 µM	[72]
	SMS-KAN	100 µM	[72]
	LA-N-6	100 µM	[72]
	SH-SY5Y	20, 50 µM	[80]
	GI-LI-N	10 µM	[74]
	NUB-7	10, 25, 50, 100 µM	[77]
	SK-N-AS	25, 50 µM	[69]
Causes DNA fragmentation	Neuro2a	50 µM	[75]
Causes membrane blebbing	Neuro2a	50 µM	[75]
Causes nuclear condensation	Neuro2a	50 µM	[75]
**Downregulates ERK 1/2 (protein)**	Neuro2a	25 µM	[75]
Upregulates caspase 9 (protein)	Neuro2a	25 µM	[75]
Upregulates caspase 3 (protein)	Neuro2a	25 µM	[75]
	GI-LI-N	10 µM	[74]
**Inactivates/cleaves PARP-1 (protein)**	Neuro2a	25 µM	[75]
	SK-N-AS	25, 50 µM	[69]
Upregulates Bex1 (mRNA)	Neuro2a	10, 25 µM	[75]
Upregulates Bex3 (mRNA)	Neuro2a	25 µM	[75]
Upregulates Bex4 (mRNA)	Neuro2a	10, 25, 50 µM	[75]
Upregulates Bex6 (mRNA)	Neuro2a	10, 25, 50 µM	[75]
Upregulates p53 (mRNA)	SH-SY5Y	5, 10, 20, 50 µM	[80]
Activates/phosphorylates p53 (protein)	Neuro2a	25 µM	[75]
Upregulates p53 (protein)	NUB-7	25 µM	[77]
Upregulates p53 nuclear translocation	NUB-7	25 µM	[77]
Upregulates p21 (mRNA)	SH-SY5Y	20, 50 µM	[80]
Upregulates p21 (protein)	NUB-7	25 µM	[77]
Upregulates ROS_i_	LAN5	10, 15 µM	[68]
	SK-N-AS		[72]
	IMR-32		[72]
**Decreases MMP**	LAN5	10, 15 µM	[68]
	IMR-32	10 µM	[74]
**Downregulates Hsp60 (mRNA)**	LAN5	12.5 µM	[82]
Upregulates Hsp60 (mRNA)	LAN5	25 µM	[82]
**Downregulates Hsp60 (protein)**	LAN5	10, 15 µM	[68]
	LAN5	25 µM	[82]
**Downregulates Hsp60 ubiquitination**	LAN5	25 µM	[82]
**Downregulates Hsp60 S-nitrosylation**	LAN5	25 µM	[82]
**Downregulates HKII (protein)**	LAN5	10, 15 µM	[68]
Upregulates Bad (protein)	LAN5	15 µM	[68]
Upregulates PTEN (protein)	LAN5	5, 10, 15 µM	[68]
**Downregulates Akt (protein)**	LAN5	10, 15 µM	[68]
	SK-N-AS	25, 50 µM	[69]
**Downregulates Akt nuclear translocation**	LAN5	10, 15 µM	[68]
Upregulates FOXO3a (mRNA)	LAN5		[68]
Upregulates FOXO3a (protein)	LAN5	10, 15 µM	[68]
Upregulates FOXO3a nuclear translocation	LAN5	10, 15 µM	[68]
Upregulates Fas-L (mRNA)	LAN5		[68]
Upregulates Bim (mRNA)	LAN5		[68]
Upregulates p27 (mRNA)	LAN5		[68]
**Downregulates NF-κB (protein)**	Kelly	100 µM	[86]
**Inhibits NF-κB DNA binding**	SK-N-MC	50, 100 nM	[32]
	SK-N-AS	25 µM	[69]
**Reduces cell migration/motility**	SH-SY5Y	10, 20 µM	[80]
Upregulates TIMP-1 (mRNA)	SH-SY5Y	10, 20 µM	[80]
**Downregulates MMP-2 (mRNA)**	SH-SY5Y	20 µM	[80]
Upregulates Bax (protein)	NUB-7	25 µM	[77]
**Downregulates Bcl-2 (mRNA)**	SK-N-AS	25, 50 µM	[69]
**Downregulates Bcl-2 (protein)**	SK-N-AS	25, 50 µM	[69]
**Downregulates LDH (protein)**	SH-SY5Y (WT)	40 µM	[83]
	SH-SY5Y (DDP)	40 µM	[83]
**Downregulates TPI1 (protein)**	SH-SY5Y (WT)	40 µM	[83]
	SH-SY5Y (DDP)	40 µM	[83]
**Downregulates GAPDH (protein)**	SH-SY5Y (WT)	40 µM	[83]
	SH-SY5Y (DDP)	40 µM	[83]
**Downregulates ENO1**	SH-SY5Y (WT)	40 µM	[83]
**Downregulates ALDOA**	SH-SY5Y (WT)	40 µM	[83]

## 5. Synergistic and Combinatorial Effects of Curcumin with Natural and Conventional Therapies for the Treatment of NB

### 5.1. Curcumin Applied Simultaneously with Other Natural Substances

While curcumin is independently effective in suppressing NB, it also exhibits synergistic effects in conjunction with several conventional and natural therapies (Table 2). Compared to other natural substances, curcumin and berberine (BBR) together demonstrate more significant cytotoxic effects on NB cells than either substance alone [90]. BBR, a plant-derived alkaloid, is a common dietary supplement. However, some clinical trials reported adverse reactions or toxicity at high doses, with 14.3% incidence of adverse effects at dosages of 0.9 to 1.5 g/day and 10.6% incidence at 1.5 g/day [91]. Therefore, while the application of 100 µM BBR is possible in vitro, clinical attainment of such a high concentration may induce potentially dangerous side effects.

In another study, SH-SY5Y cells were treated with a combination of curcumin and piperine. Cytotoxicity analysis demonstrated that increasing concentrations of curcumin- and piperine-loaded zein–chitosan nanoparticles (CPZChN; 5, 10, 25, 50, and 100 µg/mL) decreased the viability of the NB cells from 115 to 99, 30, 8, and finally 3%. CPZChN, with a mean particle size of approximately 500 nm and high encapsulation efficiencies for curcumin (89%) and piperine (87%), constitutes a promising experimental approach [92].

### 5.2. Curcumin Applied Simultaneously with Conventional Chemotherapeutics

**Figure 3:**
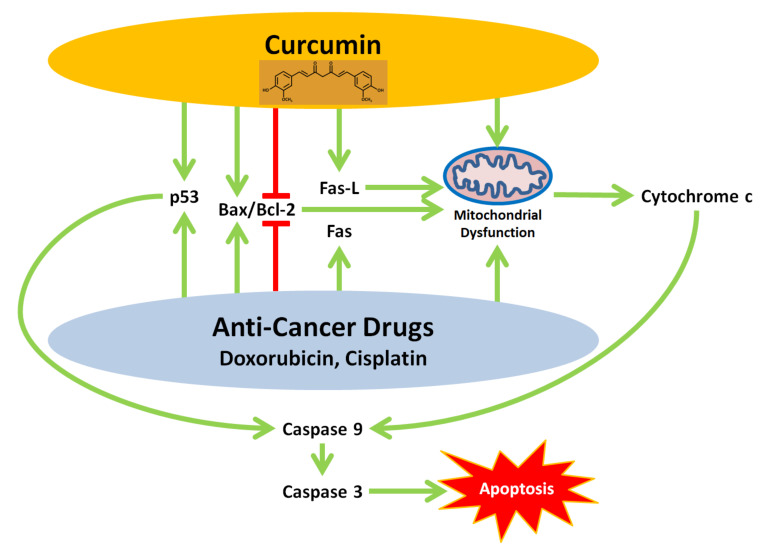
Oncologic signaling pathways are shared by curcumin and the anti-NB cancer drugs doxorubicin and cisplatin. Curcumin, doxorubicin, and cisplatin induce mitochondrial dysfunction through the upregulation of Bax and downregulation of Bcl-2. While curcumin also induces the Fas signaling pathway, doxorubicin does not, since it does not upregulate the ligand needed by the signaling pathway to proceed (Fas-L). Ultimately, the pathways lead to the activation of caspases and apoptosis.

Another potential application of curcumin in NB therapy is in combination with conventional chemotherapeutic drugs. These drugs, particularly cisplatin (a platinum compound) and doxorubicin (an anthracycline), share several anti-NB pathways with curcumin (Figure 3). Similarly to curcumin, cisplatin downregulates Bcl-2 (in SH-SY5Y cells) and upregulates Bax (in B50 cells), thereby inducing mitochondrial dysfunction [93,94]. Moreover, both curcumin and doxorubicin upregulate components of the Fas signaling pathway. Curcumin upregulates Fas-L and induces the Fas signaling pathway in LAN5 cells [68]. Doxorubicin, in contrast, upregulates Fas but not Fas-L in SH-EP-1 and SK-N-AS cells [95]. Therefore, an interesting contrast emerges, as while curcumin enhances mitochondrial dysfunction via the Fas pathway, doxorubicin does not because said pathway does not proceed without the ligand (Fas-L). Mitochondrial dysfunction leads to the release of cytochrome c, as observed in SK-N-SH cells treated with doxorubicin and B50 cells treated with cisplatin [94,96]. Furthermore, in another pathway shared with curcumin, cisplatin upregulates p53 in SH-SY5Y cells [93]. Finally, curcumin, cisplatin, and doxorubicin all induce the activation of caspases 9 and 3, leading to apoptosis.

Synergistically, curcumin potentiates the cytotoxic and pro-apoptotic effects of doxorubicin in SH-SY5Y and SK-N-AS cells [69,80]. This effect may be of clinical relevance, since it occurred at relatively low doses (0.05 µg/mL; 0.5–1.5 µM) of doxorubicin, which may feasibly be obtained in physiological environments. Moreover, Fonseka et al. (2020) evaluated a combination treatment of curcumin with doxorubicin in in vitro and in vivo NB models. The in vitro and consequent in vivo results demonstrate the oncostatic efficacy of this drug combination. Notably, curcumin and doxorubicin increased the survival and reduced the tumor volume of mice implanted with SK-N-Be(2) cells [97]. Curcumin also enhances the cytotoxic effects of 15, 30, and 45 µM cisplatin [69]. Similar drug concentrations are physiologically achievable via cisplatin infusion; as such, curcumin–cisplatin therapy has potential clinical applicability [98].

### 5.3. Curcumin in Conjunction with Radiation Therapy

Finally, curcumin also enhances the cytotoxic, apoptotic, and anticolonigenic effects of radiation therapy on NB in vitro. Potential side effects of radiation therapy include the induction of antiapoptotic genes such as Bcl-2 and the suppression of pro-apoptotic effectors such as caspases 3 and 7. However, curcumin can effectively attenuate these negative effects. Importantly, inflammation plays a central role not only in tumor resistance, but also in the incidence of side effects following radiotherapy [99]. Curcumin is not only a radiosensitizer for various malignancies, but also a radioprotector for normal tissues. As such, it exerts protective effects against the following radiotherapy toxicities: dermatitis, pneumonitis, cataractogenesis, neurocognition, myelosuppression, secondary malignancies, and mucositis or enteritis [100]. Moreover, curcumin modulates and downregulates the radiation-activated genes of the TNF superfamily, as well as the expression of NF-κB- and NF-κB-mediated pro-survival signaling in NB cells [32]. Overall, the synergistic potential of curcumin for treating NB is promising based on the results of primary studies, but is not yet supported by clinical trials.

Once sufficient basic and preclinical studies have been conducted, clinical trials will be necessary to substantiate both the isolated and synergistic effects of curcumin on NB. The combination of curcumin with radiation therapy is a promising avenue of investigation, as a low dose of 100 nM curcumin promotes apoptosis and reverses the detrimental side effects of radiation [32]. Significantly, curcumin-mediated radiosensitization and downregulation of radiation-induced antiapoptotic effectors have already been demonstrated in prostate and colorectal cancers [37,101]. Curcumin notably exerts protective effects against radiation-induced inflammation and consequent lung damage [102]. In fact, curcumin was clinically investigated for its anti-inflammatory, anticarcinogenic, and free radical scavenging properties [44].

**Table 2:** Synergistic anticancer effects of curcumin and other natural substances, conventional chemotherapeutic drugs, or radiation therapy on NB cell lines. While solid lipid curcumin particles (SLCP) were trialed with BBR and CPZChN with piperine, pure (or “standard”) curcumin was trialed with doxorubicin, cisplatin, and radiation therapy. Downregulations, decreases, and inhibitions are in bold. “Curc.” refers to “Curcumin”, “Conc.” refers to “Concentration”, and “Subs.” refers to “Substance”.

Effect	Cell Line	Curc. Type	Curc. Conc.	Subs. 2	Subs. 2 Conc.	Source
Increases cell death/decreases viability	SH-SY5Y	SLCP	20 µM	BBR	100 µM	[90]
	SH-SY5Y	CPZChN	25, 50, 100 µg/mL	Piperine	25, 50, 100 µg/mL	[92]
	SH-SY5Y	Standard	5, 10, 20 µM	Doxorubicin	0.05 µg/mL	[80]
	SH-SY5Y	Standard	10, 20 µM	Doxorubicin	5 µg/mL	[80]
	SH-SY5Y	Standard	10 µM	Doxorubicin	1 µM	[97]
	SK-N-MC	Standard	100 nM	Radiation	2 Gy	[32]
	SK-N-AS	Standard	12.5, 25 µM	Doxorubicin	0.5, 1, 1.5 µM	[69]
	SK-N-AS	Standard	12.5, 25 µM	Cisplatin	15, 30, 45 µM	[69]
	SK-N-AS	Standard	10 µM	Doxorubicin	1 µM	[97]
	SK-N-Be(2)	Standard	10 µM	Doxorubicin	1 µM	[97]
	IMR-32	Standard	10 µM	Doxorubicin	1 µM	[97]
Increases apoptosis	SH-SY5Y	Standard	5, 10, 20 µM	Doxorubicin	5 µg/mL	[80]
	SK-N-MC	Standard	100 nM	Radiation	2 Gy	[32]
**Decreases clonal growth/colony formation**	SK-N-MC	Standard	100 nM	Radiation	2 Gy	[32]
**Reduces cell migration/motility**	SH-SY5Y spheroid	Standard	2, 5, 10, 20 µM	Doxorubicin	5 µg/mL	[80]
**Reduces tumor volume**	SK-N-Be(2) xenografts, nude mice	Standard	40 mg/kg	Doxorubicin	5 mg/kg	[97]

## 6. Challenges in the Use of Curcumin in the Prevention and Treatment of NB

The application of curcumin as a preventive and therapeutic agent in NB is contingent upon its clinical delivery in sufficient doses. While a 2008 study demonstrated statistically significant decreases in the viability of SK-N-SH, Kelly, and LAN-5 NB cells treated with 0.001 µM curcumin, contemporary studies continue to utilize much higher concentrations [86]. As such, many recent studies report anticancer effects in NB cells after exposure to around 10–100 µM pure curcumin (Table 1). While such concentrations are attainable in vitro, numerous physiological challenges may hamper their clinical feasibility.

A major disadvantage of pure curcumin is its low water solubility. Organic solvents may overcome this problem; however, these solvents are rarely appropriate (or safe) for clinical use. Therefore, numerous synthetic curcumin formulations, including curcumin–alginate conjugated micelles and whey protein nanofibril carriers, have been developed to enhance the compound’s solubility [103,104].

Beyond its low water solubility, the poor oral bioavailability and rapid metabolism and elimination of curcumin have been extensively characterized and reviewed [105]. The data in Table 3 indicate that curcumin has low oral bioavailability in humans, which is improved in murine and rat models. In Wistar rats (WR), peak curcumin concentrations after oral administration are reached slowly (6 h after ingestion) in the liver and kidney, even after relatively high doses. In contrast, intravenous (IV) injection substantially decreases the time to peak concentration in the same organs. Finally, maximal curcumin concentrations in the nervous system (brain and spinal cord) are attainable relatively quickly (sometimes in a matter of minutes) after oral, IV, or intranasal administration in WR, Institute for Cancer Research (ICR) mice (ICRM), and Sprague–Dawley rats (SDR). The rapid delivery of curcumin to nervous system organs underscores the compound’s potential value as an anticancer agent in NB. In considering clinical administration, it is essential to note that curcumin lacks adverse side effects at dosages of up to 8000 mg/day [106].

## 7. Potential Solutions for the Use of Curcumin in the Nervous System and Against NB

### 7.1. Novel Curcumin Encapsulation and Delivery Modalities

As categorized in Table 3, Table 4 and Table 5, numerous experimental curcumin formulations have been developed to improve its delivery, oral bioavailability, and in vivo absorption. These formulations are diverse and include curcumin–protein complexes, nanoparticles, nanosuspensions, and microemulsions.

#### 7.1.1. Curcumin–Protein Complexes

Curcumin–protein complexes leverage the properties of certain biomacromolecules to stabilize curcumin and optimize their delivery. Mirzaee et al. developed complexes of curcumin with bovine serum albumin (BSA), casein, and beta-lactoglobulin (β-lg). In particular, native BSA and a modified form of β-lg significantly attenuated water-mediated degradation of curcumin [89]. These curcumin-protein systems could improve the half-life of curcumin under physiological conditions, and thereby enhance its bioavailability and absorption.

#### 7.1.2. Curcumin Microemulsions

In contrast with curcumin–protein complexes, microemulsions combine curcumin with oil and water. Within these microemulsions, curcumin can be combined with Capmul mono-diglyceride of medium-chain fatty acids (MCM) only (CUR Capmul ME), or both docosahexaenoic acid (DHA)-rich oil and Capmul MCM (CUR DHA ME). The injection or intranasal delivery of either microemulsion in SDR improves brain and plasma concentrations relative to standard curcumin at 1 mg/kg [111]. DHA is an essential native component of the CNS, and as such can substantially enhance the brain uptake of curcumin.

#### 7.1.3. Curcumin Nanosuspensions

Nanosuspensions, which stabilize nanoscale droplets of curcumin with biopolymers or surfactants, constitute a further delivery modality. Examples of these formulations include D-α-tocopheryl polyethylene glycol 1000 succinate-coated curcumin nanosuspension (TPGS-NS) and Tween-80-coated curcumin nanosuspension (Tween-NS). TPGS-NS and Tween-NS exhibit rapid and complete dissolution in phosphate-buffered saline, compared to the slower and incomplete dissolution of pure curcumin. Moreover, the IV administration of these nanosuspensions increases curcumin concentrations in the plasma, brain, heart, and digestive system in comparison to standard curcumin at 10 mg/kg [112].

#### 7.1.4. Curcumin Nanoparticles

Finally, nanoparticles comprise a widely investigated category of curcumin formulations. These encapsulate curcumin or other hydrophobic substances in solid nanostructures and may be conjugated with proteins to facilitate cell uptake. Examples include cerium oxide nanoparticles (CNP-Cur), which are pH-sensitive, and therefore selectively induce apoptosis in cancerous cells with minimal disruption to healthy cells, even at high concentrations. Application of a dextran coating to CNPs (Dex-CNP-Cur) enhances their biocompatibility and controls the release of curcumin over time [72]. Similarly to CNPs, silk fibroin nanoparticles (Curc-SFN) target cancer cells without harming healthy cells. Moreover, Montalban et al. synthesized SFNs using physical adsorption and coprecipitation—methods that could feasibly be scaled-up [113].

A further curcumin formulation utilizes polylactic-co-glycolic acid (PLGA) nanoparticles. In ICRM, these nanoparticles substantially increase curcumin concentrations in the brain, spinal cord, and plasma after oral ingestion as compared to pure curcumin at the same dosage of 20 mg/kg [109]. Additional modalities of curcumin delivery utilize lipid nanostructures, including solid lipid nanoparticles (C-SLN), stearic acid nanoparticles (C-SALN), and nanostructured lipid carriers (C-NLC) [88,110]. C-SLN and C-NLC improve brain concentrations compared to standard curcumin at 4 mg/kg in SDR, with a better brain absorption of C-NLC than C-SLN [110].

### 7.2. Cell Uptake and Anti-NB Efficacy of Curcumin Formulations

Protein-conjugated curcumin nanoparticles may leverage specific cellular uptake mechanisms for enhanced delivery; cellular uptake data for some formulations are detailed in Table 4. Unfortunately, only limited data specific to NB are available. Transferrin (Tf)-conjugated solid lipid nanoparticles (Tf-C-SLN) leverage Tf-receptor-mediated endocytosis to enter SH-SY5Y cells; these nanoparticles are of particular relevance, as SH-SY5Y cells overexpress the Tf receptor. Tf-C-SLN achieves a higher cellular uptake than non-Tf-conjugated curcumin SLN (C-SLN) and curcumin-solubilized surfactant solution (CSSS) [114]. Similarly, apolipoprotein-E3 curcumin-loaded poly(butyl)cyanoacrylate (ApoE3-C-PBCA) nanoparticles enter SH-SY5Y cells via endocytosis, mediated by the overexpressed low-density lipoprotein receptor (LDL-R). As such, ApoE3-C-PBCA nanoparticles achieve higher cellular uptake than non-ApoE3-conjugated curcumin PBCA nanoparticles (C-PBCA) and CSSS [115]. Finally, in the human NB cell line SH-SY5Y695 APP, a novel curcumin formulation exhibits significantly better quality and cytotoxic efficacy due to its improved cellular uptake. This formulation is well tolerated and without side effects when administered orally [116].

**Table 4:** Cellular uptake of several curcumin formulations, including suspension, nanoparticles, and protein-conjugated nanoparticles. The protein-conjugated nanoparticles (ApoE3-C-PBCA and Tf-C-SLN) utilize receptor-mediated endocytosis to enter NB cells and exhibit greater uptake over time than curcumin-solubilized surfactant solution (CSSS) and the corresponding non-protein-conjugated nanoparticles.

Curcumin Type	Administered Dose	Cell Line	Concentration	Time	Source
CSSS	10 µM	SH-SY5Y	0.85 ± 0.21 µg/10^5^ cells	12 h	[114]
CSSS	10 µM	SH-SY5Y	0.65 ± 0.11 µg/10^5^ cells	24 h	[114]
CSSS	10 µM	SH-SY5Y	0.53 ± 0.06 µg/10^5^ cells	48 h	[114]
CSSS	10 µM	SH-SY5Y	0.8 ± 0.1 µg/10^6^ cells	12 h	[115]
CSSS	10 µM	SH-SY5Y	0.57 ± 0.11 µg/10^6^ cells	24 h	[115]
CSSS	10 µM	SH-SY5Y	0.5 ± 0.06 µg/10^6^ cells	48 h	[115]
C-SLN	10 µM	SH-SY5Y	1.4 ± 0.14 µg/10^5^ cells	12 h	[114]
C-SLN	10 µM	SH-SY5Y	1.52 ± 0.2 µg/10^5^ cells	24 h	[114]
C-SLN	10 µM	SH-SY5Y	1.59 ± 0.1 µg/10^5^ cells	48 h	[114]
Tf-C-SLN	10 µM	SH-SY5Y	2.35 ± 0.11 µg/10^5^ cells	12 h	[114]
Tf-C-SLN	10 µM	SH-SY5Y	2.4 ± 0.1 µg/10^5^ cells	24 h	[114]
Tf-C-SLN	10 µM	SH-SY5Y	2.42 ± 0.12 µg/10^5^ cells	48 h	[114]
C-PBCA	10 µM	SH-SY5Y	1.45 ± 0.1 µg/10^6^ cells	12 h	[115]
C-PBCA	10 µM	SH-SY5Y	1.52 ± 0.27 µg/10^6^ cells	24 h	[115]
C-PBCA	10 µM	SH-SY5Y	1.58 ± 0.22 µg/10^6^ cells	48 h	[115]
ApoE3-C-PBCA	10 µM	SH-SY5Y	2.4 ± 0.15 µg/10^6^ cells	12 h	[115]
ApoE3-C-PBCA	10 µM	SH-SY5Y	2.6 ± 0.21 µg/10^6^ cells	24 h	[115]
ApoE3-C-PBCA	10 µM	SH-SY5Y	2.55 ± 0.24 µg/10^6^ cells	48 h	[115]

The anti-NB efficacy of several curcumin formulations is demonstrable in primary experiments (Table 5). Some of these formulations, including C-SLN, Tf-C-SLN, C-PBCA, and ApoE3-C-PBCA, exhibit statistically significant oncologic effects at low concentrations (0.5, 1, 2, 4 µM), indicating their potential clinical viability. Interestingly, other formulations, such as cerium oxide nanoparticles (CNP-Cur) and dextran-CNP-Cur (Dex-CNP-Cur), have only been tested or demonstrate significant effects at higher (and possibly clinically inviable) concentrations of 100 and 250 µM. Finally, the ability of some formulations, including CNP-Cur and Curc-SFN, to target NB cells with minimal collateral damage will be valuable. In the meantime, clinically relevant studies continue.

**Table 5:** Anticancer effects of various curcumin formulations, including protein-conjugated nanoparticles and curcumin-protein complexes, on NB cell lines. While a variety of novel curcumin formulations successfully induce NB cell death, their specific mechanistic actions remain unclear. Downregulations, decreases, and inhibitions are in bold.

Effect	Cell Line	Delivery Mechanism	Effective Concentration/Dosage	Source
Increases cell death/decreases viability	IMR-32	CNP-Cur	100 µM	[72]
	IMR-32	Dex-CNP-Cur	100 µM	[72]
	IMR-32	C-SALN	10, 100 µg/mL	[88]
	SMS-KAN	CNP-Cur	100 µM	[72]
	SMS-KAN	Dex-CNP-Cur	100 µM	[72]
	SK-N-AS	CNP-Cur	100 µM	[72]
	SK-N-AS	Dex-CNP-Cur	100 µM	[72]
	LA-N-6	CNP-Cur	100 µM	[72]
	LA-N-6	Dex-CNP-Cur	100 µM	[72]
	SH-SY5Y	CSSS		[114]
	SH-SY5Y	C-SLN	4, 16, 32, 64 µM	[114]
	SH-SY5Y	Tf-C-SLN	4, 8, 16, 32, 64 µM	[114]
	SH-SY5Y	CSSS		[115]
	SH-SY5Y	C-PBCA	4, 32, 64 µM	[115]
	SH-SY5Y	ApoE3-C-PBCA	0.5, 1, 2, 4, 8, 16, 32, 64 µM	[115]
	SK-N-MC	Curcumin-BSA		[89]
	SK-N-MC	Curcumin-Casein		[89]
	SK-N-MC	Curcumin-β-lg		[89]
	Kelly	Curc-SFN 1		[113]
	Kelly	Curc-SFN 2		[113]
Increases apoptosis	IMR-32	CNP-Cur	100 µM	[72]
	IMR-32	Dex-CNP-Cur	100 µM	[72]
	SMS-KAN	CNP-Cur	100 µM	[72]
	SMS-KAN	Dex-CNP-Cur	100 µM	[72]
	SK-N-AS	CNP-Cur	100 µM	[72]
	SK-N-AS	Dex-CNP-Cur	100 µM	[72]
	LA-N-6	CNP-Cur	100 µM	[72]
	LA-N-6	Dex-CNP-Cur	100 µM	[72]
	SH-SY5Y	CSSS	2, 4 µM	[114]
	SH-SY5Y	C-SLN	2, 4 µM	[114]
	SH-SY5Y	Tf-C-SLN	2, 4 µM	[114]
	SH-SY5Y	CSSS	2, 4 µM	[115]
	SH-SY5Y	C-PBCA	2, 4 µM	[115]
	SH-SY5Y	ApoE3-C-PBCA	2, 4 µM	[115]
Upregulates ROS_i_	SK-N-AS	Dex-CNP-Cur		[72]
	IMR-32	Dex-CNP-Cur		[72]
	SH-SY5Y	CSSS		[114]
	SH-SY5Y	C-SLN	4 µM	[114]
	SH-SY5Y	Tf-C-SLN	4 µM	[114]
	SH-SY5Y	CSSS		[115]
	SH-SY5Y	C-PBCA	2, 4, 8, 16, 32, 64 µM	[115]
	SH-SY5Y	ApoE3-C-PBCA	2, 4, 8, 16, 32, 64 µM	[115]
**Decreases MMP**	SH-SY5Y	CSSS	2, 4 µM	[115]
	SH-SY5Y	C-PBCA	2, 4 µM	[115]
	SH-SY5Y	ApoE3-C-PBCA	2, 4 µM	[115]
**Decreases Bcl-2/Bax (mRNA) ratio**	IMR-32	CNP-Cur	250 µM	[72]
	IMR-32	Dex-CNP-Cur	250 µM	[72]
Upregulates caspase 3 (protein)	SH-SY5Y	CSSS	2, 4 µM	[114]
	SH-SY5Y	C-SLN	2, 4 µM	[114]
	SH-SY5Y	Tf-C-SLN	2, 4 µM	[114]
	SH-SY5Y	CSSS	2, 4 µM	[115]
	SH-SY5Y	C-PBCA	2, 4 µM	[115]
	SH-SY5Y	ApoE3-C-PBCA	2, 4 µM	[115]

## 8. Differential Effects of Curcumin and Curcumin Formulations on NB Cell Lines

### 8.1. Patterns in NB Cell Line Usage in Curcumin Studies

Recent preclinical studies examined the effects of curcumin on diverse human and murine NB cell lines. As enumerated in Table 1, studies utilizing pure curcumin were conducted with a wide range of cell lines, some of which exhibit MYCN amplification or 11q aberration. A similar breadth can be observed for the reviewed synergistic experiments (Table 2). However, all cell uptake data arise from a single cell line, SH-SY5Y (Table 4). Finally, the data in Table 5 indicate that studies with curcumin formulations were performed with both MYCN-amplified and non-MYCN-amplified cell lines.

### 8.2. Curcumin’s Effects on Specific NB Cell Lines

#### 8.2.1. Apoptotic Mechanisms

Curcumin exhibits a wide variety of apoptotic effects when applied to NB cell lines; however, many of the compound’s individual mechanistic effects are only documented in a few cell lines (Table 1). For example, the modulation of several mitochondrial proteins (Hsp60, Bad, and Bim), the downregulation of HKII, and the upregulation of PTEN, FOXO3a, Fas-L, and p27 are reported only in the MYCN-amplified LAN5 cell line. Induction of the Bex genes and downregulation of proliferative ERK 1/2 signaling are demonstrated only in murine Neuro2a cells. The activation of caspases 9 and 3 is an essential downstream apoptotic mechanism; however, caspase 3 activation is reported in only three NB cell lines (Neuro2a, SH-SY5Y, and GL-LI-N), and caspase 9 activation in only Neuro2a cells. Finally, reductions in cell viability and upregulation of apoptosis are broadly documented.

#### 8.2.2. Synergistic Effects

The synergistic effects of curcumin with natural substances, anticancer drugs, and radiation therapy are documented in only five NB cell lines (Table 2). At present, the synergistic efficacy of curcumin–cisplatin is confirmed only in SK-N-AS cells, while the synergy of curcumin–BBR and curcumin–piperine is limited to SH-SY5Y cells. Moreover, curcumin was trialed with radiation therapy only in SK-N-MC cells. Curcumin–doxorubicin treatment has a greater breadth of efficacy, with demonstrable effects in the MYCN-amplified and 11q-abberated SK-N-Be(2) and IMR-32 cell lines, as well as in the non-MYCN-amplified SK-N-AS and SH-SY5Y lines.

#### 8.2.3. Curcumin Formulations

Broadly, novel curcumin formulations demonstrate efficacy against a wide variety of NB types and cell lines. The breadth of cell uptake studies with these formulations is highly limited—many formulations have not yet been evaluated, while the two reviewed studies utilized SH-SY5Y cells exclusively (Table 4). Moreover, preclinical oncologic studies with these formulations yielded promising general outcomes, but few specific details and limited mechanistic insights. A large proportion of the mentioned formulations exhibit confirmed efficacy in only one NB cell line or one type of NB cell line. Curc-SFNs, for example, were only trialed in MYCN-amplified and 11q-aberrated Kelly cells, and the curcumin-protein complexes curcumin–BSA, curcumin–casein, and curcumin–β-lg were trialed exclusively in SK-N-MC cells. Moreover, the demonstrated efficacy of CSSS, C-SLN, Tf-C-SLN, C-PBCA, and ApoE3-C-PBCA is limited to SH-SY5Y cells.

In contrast, Kalashnikova et al. trialed CNP-Cur and Dex-CNP-Cur against both MYCN-amplified (IMR-32, SMS-KAN) and non-MYCN-amplified (LAN-6, SK-N-AS) cell lines, reporting that the nanoparticles exhibit greater effects on the MYCN-amplified cell lines than the non-MYCN-amplified ones [72]. These results are of particular interest, as MYCN amplification typically enhances NB tumorigenesis but does not independently lead to drug resistance [117].

## 9. Proposed Areas for Further Consideration

### 9.1. Repetition of Mechanistic Trials and Synergistic Studies Across NB Cell Lines

Specific insights on curcumin’s isolated and synergistic anti-NB activities remain relatively sparse. In particular, important apoptotic mechanisms such as PTEN, FOXO3a, and Fas-L upregulation; ERK 1/2 downregulation; Bex gene induction; several components of mitochondrial dysfunction; and caspase 9 activation have each only been reported in a single NB cell line. Among these, confirmation of Bex gene induction and ERK 1/2 downregulation is limited to murine Neuro2a cells. Similarly, synergy between curcumin and BBR, piperine, cisplatin, and radiotherapy are also each limited to one cell line. As such, cross-validation of curcumin’s effects between different types of NB cell lines (e.g., murine, human, (non-)MYCN-amplified, (non-)11q aberrated, etc.) is significantly lacking. Further experiments are therefore necessary to evaluate the generalizability of curcumin’s apoptotic pathways across NB cell lines.

### 9.2. Further Testing and Standardization of Curcumin Formulations

While curcumin formulations are promising, their safety and efficacy, as well as the generalizability of their effects across NB cell lines, merit special consideration. As discussed in Section 9.1 above, curcumin formulations should be tested on a variety of NB cell lines and types. Moreover, each research group or organization currently synthesizes formulations according to its own internal procedures. As such, these formulations may vary substantially between laboratories and even between different batches from the same laboratory. While curcumin should be further investigated as an anticancer agent in NB, it is important to achieve an agreement on one or several standardized curcumin formulations before the commencement of clinical trials.

### 9.3. Investigation of the Effects of Curcumin on NB Cell Metabolism

Given recent developments in curcumin delivery systems and their efficacy against NB cell lines in primary studies, curcumin is a promising candidate for the clinical treatment of NB. However, many more experiments are needed before the introduction of curcumin for clinical trials. In particular, the effects of curcumin on NB cell glucose metabolism merit further investigation. Cancerous cells notably exhibit altered metabolic processes, which favor aerobic glycolysis over oxidative phosphorylation; this is known as the Warburg effect and has been demonstrated in NB [84]. Curcumin suppresses the Warburg effect in lung, breast, cervical, and prostate cancer cell lines, but this has not been demonstrated in NB [85]. The literature on curcumin and glucose metabolism in NB is sparse. D’Aguanno and colleagues reported that curcumin downregulates biosynthesis, glucose uptake, glycolysis, and metabolic enzymes specific to glycolysis, such as LDH, ALDOA, GAPDH, TPI1, and ENO1 [83]. Notably, epigenetic modifications that occur during tumor progression are potentially reversible, with consequent diminishment of the Warburg effect [118,119]. These results suggest that the Warburg effect, in which decreased vascularization, nutrient deprivation, and hypoxia lead to a tumor microenvironment with low pH, is a reversible process that may be targeted by curcumin. These observations could potentially also explain the anabolic and catabolic actions of phytochemicals in healthy and tumor cells, respectively. Overall, additional studies are necessary to fully elucidate the interactions between curcumin and the Warburg effect in NB.

### 9.4. Investigation of the Effects of Curcumin on NB Drug Resistance

Cancer drug resistance is a major detrimental side effect of conventional chemotherapeutic regimens. A wide variety of cellular factors, including microRNAs, regulate NB resistance to cisplatin and doxorubicin [120,121]. Correspondingly, NB drug resistance tends to increase with the strength of chemotherapeutic regimens [117]. In this light, supplementation of such regimens with curcumin, a complementary and supportive agent, could theoretically reduce the necessary dosage(s) of anticancer drug(s), and thereby decrease cancer drug resistance. However, further combinatory trials of curcumin with anticancer drugs are necessary to characterize curcumin’s potential impact on drug resistance.

### 9.5. Clinical Trials of Curcumin in NB

Curcumin has been clinically trialed in combination with chemotherapy against several cancer types, such as breast cancer, pancreatic cancer, and chronic myeloid leukemia [122]. Although the concept is promising, no clinical trials for curcumin–chemotherapy in NB are available at this time. Moreover, in situ factors, including a tumor’s specific location within the body, the tumor microenvironment, and tumor homogeneity or heterogeneity, are not easily investigated in vitro. However, they could significantly influence curcumin’s clinical efficacy.

As an independent agent or in conjunction with radiation or chemotherapy, curcumin can theoretically be administered in very high doses. Nevertheless, patient convenience and preference could be a limiting factor in oral administration, as the safe dosage of 8000 mg per day would necessitate the swallowing of sixteen 500 mg capsules per day or an equivalent dose. Despite curcumin’s safety and lack of side effects, patients may not be comfortable ingesting such large amounts.

## 10. Conclusions and Outlook

Curcumin, a naturally occurring chemical constituent of turmeric, is a promising chemopreventive and chemotherapeutic agent for a wide variety of cancers, including NB. In physiological systems, curcumin achieves its anticancer effects through the downregulation of proliferative Akt and NF-κB signaling, induction of mitochondrial dysfunction and cytochrome c release, upregulation of p53, and caspase activation, all of which lead to apoptosis. Curcumin also demonstrates synergistic effects with other natural substances, conventional chemotherapeutic drugs, and radiation therapy against NB cells.

Clearly, curcumin, as a multitargeting agent, exhibits strong activity against established tumors, with negligible toxicity to normal cells. It, therefore, has a high potential for clinical applications. Notable shortcomings, such as single-targeting and adverse toxicity, exist for current chemo- and radiotherapy treatments for NB. As such, NB treatment should be combined with more effective or safer compounds. Current evidence indicates that multitargeted curcumin may be a safe and highly effective supplement in clinical tumor control. In combination with curcumin, lower doses of chemotherapeutic drugs and radiation could achieve high antitumor efficacy, as well as low toxicity and drug resistance. In addition, curcumin has potential as an agent for safe and effective cancer prevention, treatment, and prophylaxis after chemotherapy and radiation. As such, the compound may be relevant in preventing tumor recurrence and metastasis.

Despite curcumin’s anti-NB efficacy, many of its mechanistic and synergistic activities are only demonstrated in one or several cell lines at present. As such, further testing and cross-validation with various types of NB cell lines is necessary to elucidate the impact of risk factors such as MYCN amplification on the efficacy of curcumin treatment.

Moreover, while curcumin has the potential to attenuate NB progression and counter cancer drug resistance, its clinical viability is limited by its poor oral bioavailability, low water solubility, and rapid metabolism. Numerous novel curcumin formulations—including nanoparticles, lipid carriers, nanosuspensions, and microemulsions—have been proposed to overcome these challenges. While pure curcumin is clinically safe and has minimal side effects, further basic and preclinical trials are necessary to ensure the safety of novel curcumin formulations. Ultimately, clinical trials could support or refute the viability of curcumin as an anticancer agent in NB treatment.

## Figures and Tables

**Table 3:** Bioavailability of pure curcumin and various curcumin formulations after oral, intravenous, and intranasal delivery in humans, mice, and rats. Within the sample column, “H” refers to “humans”, “WR” to “Wistar rats”, “ICRM” to “ICR mice”, and “SDR” to “Sprague–Dawley rats”.

Administration Route	Administered Dosage	Max. Concentration	Max. Time	Sample	Source
Oral	1800 mg	2.3 ± 0.3 ng/mL	7.4 ± 1.0 h	Blood Plasma, H	[107]
Oral	500 mg/kg	83.80 ± 5.46 µg/mL	6 h	Serum, WR	[108]
Oral	500 mg/kg	490.3 ± 32.0 µg	6 h	Total Blood, WR	[108]
Oral	500 mg/kg	135.2 ± 5.26 µg	6 h	Liver, WR	[108]
Oral	500 mg/kg	9.03 ± 1.11 µg	6 h	Kidney, WR	[108]
Oral	500 mg/kg	36.19 ± 3.10 µg	1 h	Intestine, WR	[108]
Oral	20 mg/kg	2.03 ± 0.69 ng/g	15 min	Brain, ICRM	[109]
Oral	20 mg/kg	23.49 ± 11.57 ng/g	7.5 min	Spinal Cord, ICRM	[109]
Oral	20 mg/kg	0.60 ± 0.44 ng/mL	15 min	Plasma, ICRM	[109]
Oral	400 mg/kg	30.32 ± 3.10 ng/g	5 min	Brain, ICRM	[109]
Oral	400 mg/kg	129.16 ± 63.12 ng/g	5 min	Spinal Cord, ICRM	[109]
Oral	400 mg/kg	79.82 ± 49.00 ng/mL	3 min	Plasma, ICRM	[109]
Oral/PLGA-Curcumin	20 mg/kg	12.71 ± 6.63 ng/g	3 min	Brain, ICRM	[109]
Oral/PLGA-Curcumin	20 mg/kg	85.88 ± 54.27 ng/g	7.5 min	Spinal Cord, ICRM	[109]
Oral/PLGA-Curcumin	20 mg/kg	41.33 ± 16.03 ng/mL	3 min	Plasma, ICRM	[109]
Injection	4 mg/kg	0.00 ± 0.00 ng/g	-	Brain, SDR	[110]
Injection/C-SLN	4 mg/kg	114.22 ± 58.21 ng/g	0.5 ± 0.28 h	Brain, SDR	[110]
Injection/C-NLC	4 mg/kg	390.30 ± 35.93 ng/g	1 ± 0.28 h	Brain, SDR	[110]
Injection	1 mg/kg	968.11 ± 67.1 ng/mL	0.0833 h	Plasma, SDR	[111]
Injection	1 mg/kg	90 ± 6.82 ng/mL	1 h	Brain, SDR	[111]
Injection/CUR Capmul ME	1 mg/kg	1875.45 ± 91.6 ng/mL	0.0833 h	Plasma, SDR	[111]
Injection/CUR Capmul ME	1 mg/kg	120 ± 8.61 ng/mL	1 h	Brain, SDR	[111]
Injection/CUR DHA ME	1 mg/kg	2059.8 ± 103.6 ng/mL	0.0833 h	Plasma, SDR	[111]
Injection/CUR DHA ME	1 mg/kg	253 ± 18.74 ng/mL	1 h	Brain, SDR	[111]
Intranasal	1 mg/kg	31.25 ± 2.8 ng/mL	0.5 h	Plasma, SDR	[111]
Intranasal	1 mg/kg	122 ± 7.89 ng/mL	0.5 h	Brain, SDR	[111]
Intranasal/CUR Capmul ME	1 mg/kg	39.62 ± 2.3 ng/mL	0.5 h	Plasma, SDR	[111]
Intranasal/CUR Capmul ME	1 mg/kg	324 ± 22.43 ng/mL	0.5 h	Brain, SDR	[111]
Intranasal/CUR DHA ME	1 mg/kg	48.75 ± 3.1 ng/mL	0.5 h	Plasma, SDR	[111]
Intranasal/CUR DHA ME	1 mg/kg	523 ± 30.95 ng/mL	0.5 h	Brain, SDR	[111]
Injection	10 mg/kg	661.66 ± 48.604 ng/mL	-	Plasma, WR	[112]
Injection	10 mg/kg	6.6 ng/g	0.25 h	Brain, WR	[112]
Injection	10 mg/kg	50.0 ng/g	0.25 h	Liver, WR	[112]
Injection	10 mg/kg	51.6 ng/g	0.50 h	Lung, WR	[112]
Injection	10 mg/kg	24.1 ng/g	0.25 h	Heart, WR	[112]
Injection	10 mg/kg	25.7 ng/g	0.25 h	Kidney, WR	[112]
Injection	10 mg/kg	24.0 ng/g	1.00 h	Spleen, WR	[112]
Injection/Tween-NS	10 mg/kg	1311.62 ± 172.294 ng/mL	-	Plasma, WR	[112]
Injection/Tween-NS	10 mg/kg	66.7 ng/g	0.75 h	Brain, WR	[112]
Injection/Tween-NS	10 mg/kg	94.5 ng/g	0.50 h	Liver, WR	[112]
Injection/Tween-NS	10 mg/kg	282.1 ng/g	0.50 h	Lung, WR	[112]
Injection/Tween-NS	10 mg/kg	275.5 ng/g	0.25 h	Heart, WR	[112]
Injection/Tween-NS	10 mg/kg	86.5 ng/g	0.50 h	Kidney, WR	[112]
Injection/Tween-NS	10 mg/kg	79.4 ng/g	3.00 h	Spleen, WR	[112]
Injection/TPGS-NS	10 mg/kg	1121.28 ± 46.259 ng/mL	-	Plasma, WR	[112]
Injection/TPGS-NS	10 mg/kg	17.7 ng/g	0.50 h	Brain, WR	[112]
Injection/TPGS-NS	10 mg/kg	169.8 ng/g	0.25 h	Liver, WR	[112]
Injection/TPGS-NS	10 mg/kg	253.4 ng/g	2.00 h	Lung, WR	[112]
Injection/TPGS-NS	10 mg/kg	38.7 ng/g	0.25 h	Heart, WR	[112]
Injection/TPGS-NS	10 mg/kg	70.1 ng/g	0.50 h	Kidney, WR	[112]
Injection/TPGS-NS	10 mg/kg	333.0 ng/g	1.00 h	Spleen, WR	[112]

## Abbreviations

1,3-BPG	1,3-Bisphosphoglycerate
2-PG	2-Phosphoglyceric Acid
5-LOX	5-Lipoxygenase
Akt (PKB)	Akt Serine–Threonine Kinase (Protein Kinase B)
ALDOA	Aldolase
AP-1	Activator Protein 1
ApoE3-C-PBCA	Apolipoprotein-E3 Curcumin-Loaded Poly(Butyl)Cyanoacrylate (nanoparticles)
ATP	Adenosine Triphosphate
β-lg	βeta-Lactoglobulin
Bad	Bcl-2-Associated Agonist of Cell Death
Bax	Bcl-2-Associated X Protein
BBR	Berberine
Bcl-2	B-cell Lymphoma 2
Bex1	Brain-Expressed X-linked (gene) 1
Bex2	Brain-Expressed X-linked (gene) 2
Bex3	Brain-Expressed X-linked (gene) 3
Bex4	Brain-Expressed X-linked (gene) 4
Bex6	Brain-Expressed X-linked (gene) 6
Bid	BH3-Interacting Domain Death Agonist
Bim	Bcl-2-Interacting Mediator of Cell Death
BSA	Bovine Serum Albumin
C-NLC	Curcumin-Loaded Nanostructured Lipid Carriers
C-PBCA	Curcumin-Loaded Poly(Butyl)Cyanoacrylate (nanoparticles)
C-SALN	Curcumin-Loaded Stearic Acid Lipid Nanoparticles
C-SLN	Curcumin-Loaded Solid Lipid Nanoparticles
CNP-Cur	Cerium Oxide Nanoparticles–Curcumin
CNS	Central Nervous System
COX-2	Cyclo-Oxygenase 2
CPZChN	Curcumin- and Piperine-Loaded Zein Chitosan Nanoparticles
CSSS	Curcumin-Solubilized Surfactant Solution
CUR Capmul ME	Curcumin Capmul MCM Microemulsions
CUR DHA ME	Curcumin Docosahexaenoic Acid-Rich Microemulsions
Curc-SFN 1	Curcumin-Loaded Silk Fibroin Nanoparticle Preparation 1 (physical adsorption)
Curc-SFN 2	Curcumin-Loaded Silk Fibroin Nanoparticle Preparation 2 (coprecipitation)
DDP	Cisplatin-resistant
Dex-CNP-Cur	Dextran–Cerium Oxide Nanoparticles–Curcumin
DHAP	Dihydroxyacetone Phosphate
DNA	Deoxyribonucleic Acid
ECM	Extracellular Matrix
EMT	Epithelial–Mesenchymal Transition
ENO1	Enolase 1
ERK	Extracellular-Signal-Regulated Kinase
Fas (CD95)	Fas Cell Surface Death Receptor (Cluster of Differentiation 95)
Fas-L	Fas Ligand
FBP	Fructose 1,6-BiPhosphate
FLIP	FLICE Inhibitory Protein
FOXO3a	Forkhead Box O3a
G3P	Glyceraldehyde-3-Phosphate
GAPDH	GlycerAldehyde-3-Phosphate Dehydrogenase
Gy	Gray
HIF-1α	Hypoxia-Inducible Factor 1 αlpha
HKII (HK2)	Hexokinase II (Hexokinase 2)
Hsp60	Heat Shock Protein 60
ICR	Institute for Cancer Research
ICRM	ICR Mice
IL-1	Interleukin 1
IL-6	Interleukin 6
iNOS	Inducible Nitric Oxide Synthase
LDH	Lactate Dehydrogenase
LDL-R	Low-Density Lipoprotein Receptor
MMP	Mitochondrial Membrane Potential
mRNA	Messenger Ribonucleic Acid
MYCN	N-myc Proto-oncogene
NB	Neuroblastoma
NF-κB	Nuclear Factor κappa Light-Chain Enhancer of Activated B-cells
p21 (CDKN1A)	(Cyclin-Dependent Kinase Inhibitor 1A)
p27 (CDKN1B)	(Cyclin-Dependent Kinase Inhibitor 1B)
p53	Tumor Protein p53
PARP-1	Poly(ADP-Ribose) Polymerase 1
PEP	Phosphoenolpyruvate
PLGA	Polylactic-co-Glycolic Acid (nanoparticles)
PTEN	Phosphatase and Tension Homolog
ROS	Reactive Oxygen Species
SDR	Sprague–Dawley Rats
SLCP	Solid Lipid Curcumin Particle
Tf	Transferrin
Tf-C-SLN	Transferrin-Conjugated, Curcumin-Loaded Solid Lipid Nanoparticles
TNF-α	Tumor Necrosis Factor αlpha
TPI1	Triosephosphate Isomerase 1
Tween-NS	Tween 80-Coated (Curcumin) Nanosuspension
WR	Wistar Rats
WT	Wild-Type
